# The Ontology of Vision. The Invisible, Consciousness of Living Matter

**DOI:** 10.3389/fpsyg.2016.00089

**Published:** 2016-02-23

**Authors:** Giorgia Fiorio

**Affiliations:** Ca' Foscari University of VeniceVenice, Italy

**Keywords:** presence in visual perception, human archetype, living matter, inner consciousness, semantic memory

## Abstract

If I close my eyes, the absence of light activates the peripheral cells devoted to the perception of darkness. The awareness of “seeing oneself seeing” is in its essence a thought, one that is internal to the vision and previous to any object of sight. To this amphibious faculty, the “diaphanous color of darkness,” Aristotle assigns the principle of knowledge. “Vision is a whole perceptual system, not a channel of sense.” Functions of vision are interwoven with the texture of human interaction within a terrestrial environment that is in turn contained into the cosmic order. A transitive host within the resonance of an inner-outer environment, the human being is the contact-term between two orders of scale, both bigger and smaller than the individual unity. In the perceptual integrative system of human vision, the convergence-divergence of the corporeal presence and the diffraction of its own appearance is the margin. The sensation of being no longer coincides with the breath of life, it does not seems “real” without the trace of some visible evidence and its simultaneous “sharing”. Without a shadow, without an imprint, the numeric copia of the physical presence inhabits the transient memory of our electronic prostheses. A rudimentary “visuality” replaces tangible experience dissipating its meaning and the awareness of being alive. Transversal to the civilizations of the ancient world, through different orders of function and status, the anthropomorphic “figuration” of archaic sculpture addressees the margin between Being and Non-Being. Statuary human archetypes are not meant to *be visible*, but to *exist* as vehicles of transcendence to outlive the definition of human space-time. The awareness of individual finiteness seals the compulsion to “give body” to an invisible apparition shaping the figuration of an ontogenetic expression of human consciousness. Subject and object, the term “humanum” fathoms the relationship between matter and its living dimension, “this de facto vision and the ‘there is’ which it contains.” The project reconsiders the dialectic between the terms vision–presence in the contemporary perception of archaic human statuary according to the transcendent meaning of its immaterial legacy.

## Introduction

As if absence was a volume inhabiting the space around us, returning home and, say, noting the arrangement of objects, identical, albeit now bathed in a different shade of light, who hasn't faced one day a place suddenly unrecognizable? For reality is a *before*-ness and an *inside*-ness—“thought through my eyes” (Joyce, 2012)—that resides within the observer's vision.

If I close my eyes, the absence of light activates the peripheral cells devoted to the perception of darkness (von Helmholtz, 1962; Gregory, 1990). The awareness of “seeing oneself seeing” is in its essence a thought, one that is internal to the vision and preceding any object of sight. To this amphibious faculty, the “diaphanous color of darkness,” Aristotle (1907) assigns the principle of knowledge (Aristotle, 1907; Agamben, 2005). In Muḥiddīn Ibn ‘Arabī’ (1981) neat metaphor, the outer rind and inner stone of a fruit (*El-Qishr; wa'l-Lobb*) or, in other words the dynamic binding each point of the circumference to its permanent principle of irradiation, the center (Guénon, 1995).

“Vision is a whole perceptual system, not a channel of sense” (Gibson, 1986a).

The *head-eye* structure conducts thoughts and acts from the summit of a body whose erect posture is orthogonal to the surface of the Earth onto where it stands. Functions of vision are interwoven with the texture of human interaction within a terrestrial environment (Gibson, 1986b) that is in turn contained into the cosmic *order*.

The sensation of *being*, the here and now, no longer coincides with the breath of life, it does not seems “real” without the trace of some visible evidence and its simultaneous “sharing.” Without a shadow, without an imprint, and destined for multiple invisible witnesses, the numeric *copia* of the physical presence inhabits the transient memory of our electronic prostheses. As information anticipates and alters the flow of events, the indefinite plethora of news erase them one after another; in the same way, a rudimentary “visuality” (Crary, 2013) replaces tangible experience dissipating its meaning and the awareness of being alive.

## Ontological structure

In ancient times, the *symbolon* of Mysteries is the accidental fracture of a primordial whole: a stone or a stick broken and then re-joined, wherein the re-conjunction of the divided elements authenticates their relationship.

As we know, the universal character of Being, in its neutral plural form, *ta ónta* (from ἔιναι, to be), does not express multiple realities, but rather the wholeness, like Heidegger's *Seiende*, of *that which is*. As “principle of the manifestation” (Guénon, 2001), the unity of Being is suspended in the dual margin, between the undivided flow of internal sense and that of its ever-changing manifest refraction.

In the perceptual integrative system of human vision, the convergence-divergence of the corporeal *presence* and the diffraction of its own *appearance* is the margin.

*The inside of an outside that is the outside of the inside* (Merleau-Ponty, 1964; Johnson, 1993) joins and splits in a *chi*-shaped (χ) anatomical formation named *chiasm* (from *chiázō*, χιáζω, to cross, to go through) set in the cranial cavity, where the optic nerves cross and join the visual impulses.

For Plato (2007) the “Soul of the world,” that regulates the motion of the universe intersects two circles in the figure of a *chi* (χ) traced by the Demiurge, wherein the obliquity of the Ecliptic represents the axis of the Other with respect to the Equator, which is the axis of the Self. By definition without a subject, the reflexive relation encompasses necessarily the presence of another term, it-*self*.

The particle *se*, (Gr. *he*, Lat. *sē*, Skr. *sva*) refers to something that is *habitual* and, at the same time, *separate*. The term, itself-*heauton* contains both the relation of an endless parting and that of a return (Agamben, 2005)—*ēthos anthrōpō daimōn*—(Heraclitus: Diels and Kranz, 1903). According to the doctrine of *Ittyḥād* (unification), “my very separation is my union” (Ibn ‘Arabī, 2006).

Within the optic array of the Earth, the “relation of location is not given by degrees of azimuth and elevation (for example) but by the relation of inclusion” (Gibson, 1986c). Each second of an hour, each year in a century over the millennia, each grain of sand of each beach of a country of a continent are all embedded *one* into another according to their proportions of size or duration. A transitive host within the internal resonance of an inner-outer environment, the human being is the contact-term between two orders of scale—molecular and cosmic—both bigger and smaller than the individual unity (Simondon, 2005). “My” infra and ultra-corporeal experience of the world embodies its “Double” (Vitiello, 2001).

*Tat tvam asi*, “*this* you are” (Chāndogya Upanishad, 1921), or, as according to the inscription once carved in the pronao of the Temple of Apollo at Delphi, gnōthi seautón, “know thyself”. *Gnōthi seautón*.

The self-reflective shift inhabits the world since the beginning knowledge. The dual relationship between the eye and the brain, between the *Eye, Hades* (*"A*ιδης) and its *Pupil, Kore–Hestía* (*Kóρη*—‘*Iστíη* or ‘*Eστíα*), between the two *birds* of the Vedas on the same branch of the tree: “One of them eats the sweet berry of the *pippal*; the other, without eating, watches” (Calasso, 2014a). The rooting of such recognition, according to Simone Weil, is where the past transmits itself alive to future generations (Weil, 2014); looking backwards, it is the vector decoding the memory of meanings.

While for the Western culture the significance of vision is interwoven with that of knowledge, the archaic and Oriental principle associates the nexus of retinal perception and the counter impulse from the visual cortex to the cosmic *respiration*.

According to Homer, the human being sees through the mind or the lungs and perceives the visual imagination through breath, *thumos* (*θυμóς*, *ὄσσoντo* *θυμῷ*; Onians, 1951a). An aerial substance, the *thumos* is for the Ancients inhabited by its liquid principle condensing—*like dew*—the heat of the blood. An attribute of the consciousness residing in the breast of the living being, the *thumos* abandons the *white bones* of the expiring creature. *psychē* is the *soul-breath*, the vehicle carrying the principle of Life itself. Homer identifies *psychē* with shadows, *skiά* (Butler, 1900); as Pindar refers (Pindar, 1997), when Death overtakes the human being, in the Reign of Hades the “*aiōnos eídōlon*”—that is the soul, the *shadow* of Life—survives (Onians, 1951b).

The *pupil* is not only the messanger conveying visual data from/to the fovea and the primary cortex, it is the contact between visible and invisible worlds.

*Kore*, the *Pupil*, symbol of fertility and life, embodies the simulacrum of the Earth's vital principle—*Hestía* (Porphyry, 1993). First daughter of Cronus (*K*ρóν*o*ς) and *Rea* (*Ṕ*εα)—in the ‘physics’ of Stoics—*Hestía* is the immutable *heart* of the Earth, the *center* for Plato and permanent *essence* of things (Plato, 1892a), that is in turn the foundation of Cosmos (Plato, 1892b; Macrobius, 2011).

The Upanishads' vital breath (*prāna*) envisages all single perceptions as a unified whole. “*Brahman* is breath, *Brahman* is happiness (*ka*), *Brahman* is space (*kha*) […].” “Brahman is *kha*, space; space is primordial, space is windswept,” “That which is called *brahman* is this space, ā*kāsa*, which is outside man. This space which is outside man is the same as the one within man…”(Calasso, 2014a). The *ātman*, that is the human soul, has the Universe as a body (Weil, 1956). In China, ideograms are vehicles of *apparition* where the spiritual expression and the true meaning of reality gives “form” to the “circulation” of the universal breath (Lagerwey, 2003).

## Inner vision and presence

The term *mystērion*—related to the meaning “to initiate” (μυε$\tilde{i}$ν) and to that of “closing the eyes” or “the lips” ($\mu \overset{´}{\upsilon }\varepsilon$ιν)—refers to that which cannot be expressed. Celebrations of *Mysteria* began in Eleusi when the veiled *mystes* ($\mu \overset{´}{\upsilon }\sigma \tau \eta \varsigma$) closed the eyes to *enter* in the darkness plunging into his/her own unutterable intimacy. Romans named such “closure” and “entrance” into shadows *in-itia*. *Mysteria* were about trespassing the limit from where origins of the esoteric experience and metaphysical knowledge unveiled their unspeakable brightness. The apparent paradox of public celebration of *Mysteria* underpinned the inexpressibility of living phenomena as events belonging both uniquely to the subjective experience of the *single* and to the universal one (Kerényi, 1979).

There are two types of substitution: by equality or by identity. It is the difference between the shadow and the mirrored image: if the latter is the same in every mirror, the subject does not necessarily recognize his/her own identity in the respective reflection. Conversely, like the footprint in the sand, the shadow before or after each one of my steps is always and only mine (Heidegger, 2002). As the dislodged shard of rock represents the mountain or, as according to Mauritius Cornelius Escher, one single fish's scale encompasses the *specie* (*Fishes and scales* lithograph, 1959; Hofstadter, 1980), the qualitative character of identity embodies an absolute Otherness.

In a comparison of *image* and *figure*, from the root *ajem-*, to imitate, the *imago* of ancient Rome does not express an order of the *idea* but rather the substantial transcription (according to Pliny the Elder) of the *maxima similitude* “expressed” in wax, *expressi cera uultus*. It is the matrix of the transmutation of matter literally “taking shape” in the contact with the face itself of the dead subject, subsequently transferred to its opposite convex double in plaster (Didi-Huberman, 2000).

From the Greek *skhêma* (*σχημα*, shape, form) and from the Latin *fingere*, for molding, the “figure” denominates the aspect of an abstract model, it is the interior representation of what is not defined by reality: the acknowledgement of an apparition that *figures*—regardless—the actual presence.

For as we know, “matter is in itself not a reality but only a possibility, a ‘potentia’; it exists only by means of form” (Heisenberg, 1958).

In optical terms an image is the refraction of an object produced by a reflecting device. *An array considered as a structure* or an *arrangement of invariants of structure* for Gibson (1986d); a *neural configuration* or *map* for Damasio (2010) defined by the *interrelation* between its parts, the mental image is a self-reflective configuration, expanding or contracting the initial ordainment according to a universal *form* of which *model is internal within you* (Philo, 1854; Saint Thomas Aquinas, 1947–8).

In the composite articulation of the hiatus between the action of the motion system and the sensory awareness, “the voluntary act begins—according to Libet—before the conscious will to act” (2004). Gilbert Simondon, in his general hypothesis of the genesis of images (2014), relates the evolutionary process internal to the vision to three phases, which are characterized by a self-kinetic enactment that is oriented in accordance with different degrees of awareness. In the first phase of intuition and anticipation occurs an endogenous impulsion, which is impressed in a molding that harks back to the phylogeny of Being. In ancient times, the *numinous* character determined the appearance invading the subject's imagination with a relative independence from its conscious and unified activity.

Lucretius (2008) suggests there are *simulacra* penetrating the liminal parts of the soul—*per rara cientque tenvem animi naturam*—and entirely invading the human subject.

As an impulse trigger, *desire* elicits a bundle of *pre-optive* motor tendencies that convey the inner-vision toward pre-visualization and the construction of frames of actions that are at the limit of consciousness. In this gestation converges the entire duration of the human being's internal activity and that of the environment in which its existence unfolds. Thus, the actual sensorial perception is ruled by the dialectics of contact with the innate structures of the ancestral memory. What follows is a mental systematization of the imagined reality where the subject, appropriates an “analogon” of the world (Simondon, 2014). Such *anologon* is for Freeman and Vitiello a “coherent, highly textured brain activity pattern,” the “Double.” The “Double is the Mind” in its capillary entanglement with brain-matter. “Brains” are *open* dynamical systems where the Double, within the *many-body* dissipative model projects continuous time-reversal *pre-figurations* and “imagines” the world it produces as “hypotheses and predictions that we experience as perception” (Freeman and Vitiello, 2006, 2010, 2015).

**Figure 1:**
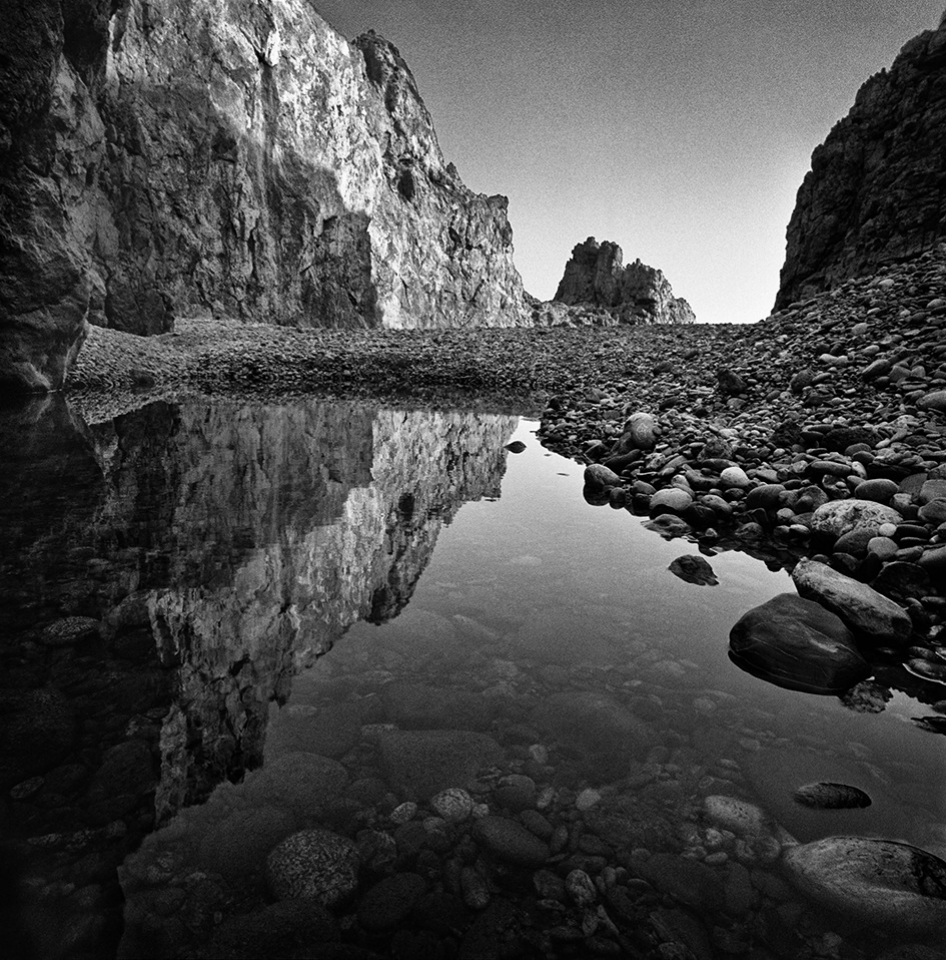
**Giorgia Fiorio © 2010 ***Kirakoulou Faranghi***, Kythira Island, Greece**.

The memory does not follow, rather it foresees and guides the—*in-tention*—thread of sensorial perception within an inner-outer interwoven environment (Figure 1). “Visual perception is not a passive recording of stimulus material but an active concern of the mind” (Arnheim, 1969a). *Evolutive*, cognitive activity unfolds within itself in the articulate pattern of a transparent architecture connecting the self-referential innate structures of the subject's to his/her immediate phenomenal relation to the world (Changeux, 2004).

## Intention and meaning

Transversal to the civilizations of the ancient world, through different orders of function, proximity and *status*, the anthropomorphic “figuration” (Vernant, 1990, 2003) of archaic sculpture questions the “idea of Being” plunged into the different cultural and ritual environment where human ideotypes begin to appear.

Ancient statues do not come to light in order to be *visible* as artworks, but rather to *exist* (Benjamin, 1936) as vehicles of transcendence. Archaeological heritage is the expression of a metaphysical condition that projects itself beyond manifest definition to outlive the individual space-time (Vernant, 1996a).

The “body” of Gods, intangible and present as an ever-changing immanence, is the absolute transcendence in the wholeness of unity.

The human body, this first and last object of any human knowledge, inhabits the temporal measure of its corporeal definition (Heidegger, 2005). Abode of a flow of impulses and breaths, this body is destined to dissipate again in the indeterminable-ness of reality, yet, at the threshold of its temporal measure, only its own utter one-ness inhabits it (Vernant, 1996b). In its motionless wholeness, the archaic human statue embodies the mobile complexity of living phenomena within the endless transfiguration of the space that it occupies (Figure 1). As the invisible archetype of “something that is not,” the archaic *ideo-type* of the human figure is certainly not that of our contemporary perception, not an “idol” exclusively *seen* in the static *actuality* of its visible evidence, bereaved of the transcendent spectrum of its function and meaning (Porphyry, 1993).

Shadow of its own frame the Greek *eídōlon* is a margin between *Being* and *Non-Being*, wherein “the body is an image of the soul, which is called its form” (Coomaraswamy, 1997). The statuary archetype of the human soul, that is the shadow—the Greek *eídōlon, Ka* for the Egyptians (Maspero, 1893; Stoichita, 1997)—embodies the wholeness of an internal figure in the transfiguration of its aspect, unique and different, within the living reflection of each viewer's vision. The awareness of individual finiteness seals the compulsion to “give body” to an invisible apparition shaping the archaic statuary figuration as an ontogenetic expression of human consciousness.

Intrinsic to the essential quality of *life-pulse* (Bergson, 1914) consciousness embodies and altogether transcends its own corporeal definition. Mobile and arbitrary, the phenomenal localization of consciousness belongs to the unconditioned character of *volition*; removed from sensation or perception, its activity proceeds according to Jaynes (1995) by “diachronic” processes of interpolation. Within the curve of the process informing the contact between *intention* and *finality*, in Sanskrit one word defines both terms “meaning” and “utility”: â*rtha* (Coomaraswamy, 1986). The term *télos* in the sense of “conclusion,” encompasses the multiplicity of its sematic determinations within one whole continuous move (Onians, 1951c). Such as the invisible crowning of destiny, volition (*voulisis*; Plato, 1892c) circumscribes the essence of purpose as oriented to the finality of its unfathomable meaning.

In accord with the *hylomorphic* principle of Aristotle's (1933) *sýnolon*, whereby substance is the indwelling form of which matter is composed, the “individuation” resides for Simondon in an incessant process of *actualization* of matter into a form. If the physical existence *ends* at its limits, the living being *is* always contemporaneous to itself.

We know the *substantia*, from *sub stare*, is “that which stands beneath,” the *substratum* (support) of universal manifestation and we know matter, *ūly (ὔλη)* is the hidden vegetative principle, the *root* from where the Being draws the lymph of any manifest animation (Guénon, 2002).

The *individuation*—that is Life—takes place in a continuous internal resonance of the human constitutive structure within its own concentration (Simondon, 2005).

*Thymisou sōma* (*body, remember*): toward the end of his life, the imperative of Cavafy (1992) poignantly invokes that of the flesh.

## Observation and “information”

Living phenomena when being observed are altered, for what is observed is not the essence of reality, but rather the reality of what is observed. “To observe,” from the Latin, *ob-servare*, means to adapt.

Modern “civilization,” raised upon the cult of its patent *visibility*, of which the *secular society* is the corner-stone, is the first in history to exclusively project itself onto its own *immanent existence* (Calasso, 1994, 2014a,b). “You are about to enter a world where the recording of an event eclipses the event itself,” anticipated Joseph Brodsky 25 years ago (Brodsky, 1995).

The invisible presence intrinsic to origin's *recognition* being superseded—extraneous to and separated from the internal-Self—the modern *individual-observer* no longer inscribes his/her subjective experience in the world, but rather in the incessant reception of data whereas the indefinite interface of the network unhinges the temporality and function of spaces. For an increasing number of people most activities are conducted by passive reception of stimuli; numberless inputs ‘organize’ the arc of our actions, inputs that rule and on our behalf shred in fragments the internal order in the flow of our days and dissipate any spontaneously *organized* activity or skill.

In the process of conforming an ancestral scheme of perception to new cognitive schemes and behavioral structures, the loss of visual attention is the peak moment of a vast genealogy, a genealogy wherein the overturning of visual experience begins with the passage from geometrical to physiological optics (17th and 18th centuries).

As extensively outlined by Crary (1992), to understand the fission between the internal Being and the projection of the individual onto the world it is necessary to examine the causes of the reversal of terms in the relation *presence-vision* during the last two centuries. In the visual culture of the 19th century, the new optical equipment applied to new forms of mass entertainment introduced realistic effects that were based on a radical abstraction and reconstruction of the visual experience. Thus, removed from the incorporeal relations of the *camera obscura*, the visual phenomenon is subsequently re-located in the human body. The rise of new production and disciplinary needs generates the necessity of parameters that enable the study of individual behaviors, in which the observer becomes object of observation, experimentation and *normalization* (Crary, 1992). Throughout the 20th century increasingly passive and instrumental forms of attention codify the activity of the eye through unaware responses (Benjamin, 1955) to stimuli, leading to the progressive eclipse of the immediate surroundings.

“Man's signs and structures are records because, or in so far as, they express ideas separated from, yet realized by, the process of signaling and building” (Panofsky, 1970). The multiplication of signs and the dematerialization of images generate perceptive dimensions transcending human wavelength wherein the presence of the observer no longer corresponds to its position in space.

Today's simultaneousness is a make-shift replacing the visual presence with “parallel temporalities” of illusory forms of human interaction and “social integration” (Crary, 1992, 2001). The awareness of the “bio-deregulation” (Brennan, 2003) induced by the perceptual leveling of dependency on information and communication systems that the technological imperative imposes (Stiegler, 2009) is the first step toward the preservation of the Freedom of the person, the human heritage of cultural identity and historical memory.

## Project *humanum*®: the archaeology of light

The project reconsiders the dialectic between the terms *vision–presence* in the contemporary perception of the figuration of archaic human statuary according to the transcendent meaning of its immaterial legacy.

As both object and subject the term *humanum* addresses the relationship between matter and its living dimension, “this de facto vision and the ‘*there* is’ which it contains” (Merleau-Ponty, 1964). The statue exists as a tangible form of the invisible. Its aspect, intrinsic to a latent transformation, is an *attribute of my perception* (Merleau-Ponty, see Johnson, 1993), it does not participate in the definition of its volume if I am able to envisage its *different relief* (Bergson, 1934) in a photograph, a planar surface by definition. *Phôs-graphı, light-script*, a scalpel to carve shadows whereas *mimesis* only casts the “sign” of an internal identification, photography can no longer be intended as a replica of what is already a reflection.

**Figure 2:**
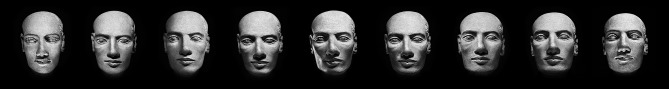
*****Head of Man*** Ca. 2289, known as ***Tête Salt***, painted limestone, estimated Middle Empire 18th–20th century BC possible provenance Karnack, High Egypt Department of Egyptian Antiquities Musée du Louvre, Paris, France, ***Humanum***® ***Paradeigma*** Frieze ***F9*** dimension 1 to 1, 27.54 × 207 cm, Giorgia Fiorio © 2015**.

**Figure 3:**
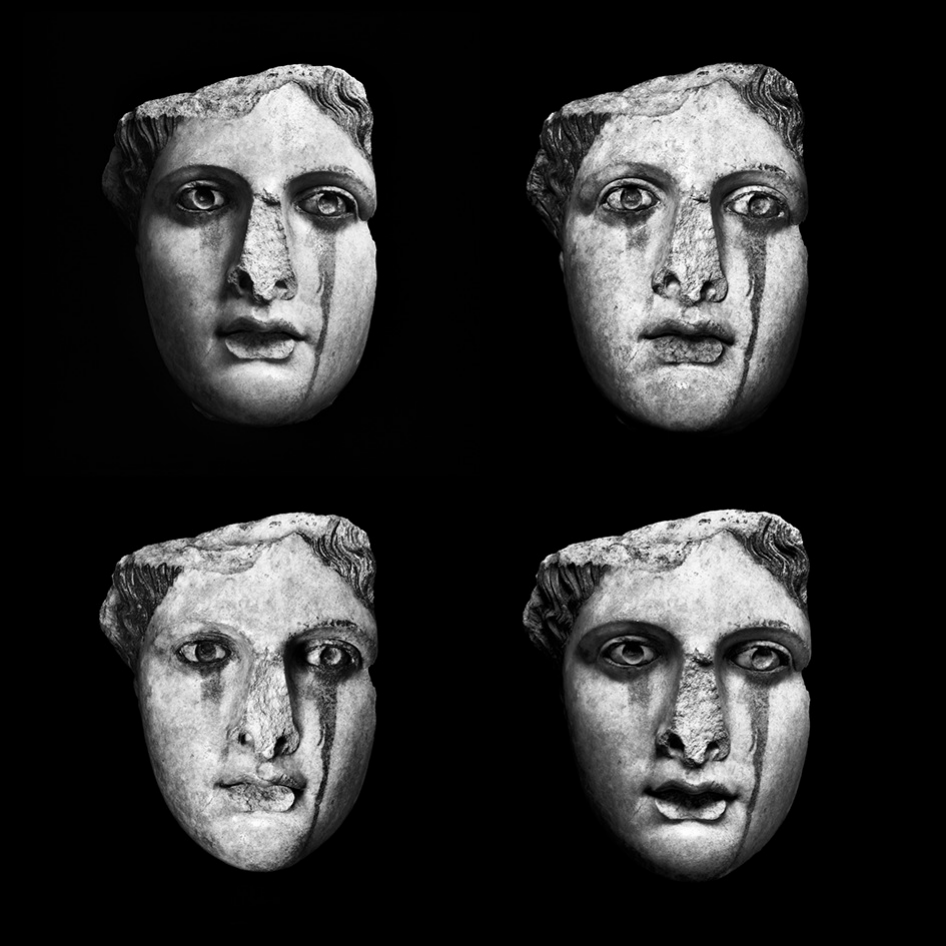
*****Female Head*** Nam. 244, Parian Marble, 2nd CE Acropolis Museum of Athens, Greece, ***Humanum***® ***Paradeigma*** Square ***Q4*** dimension 1 to 1, 81 × 81 Giorgia Fiorio © 2012**.

**Figure 4:**
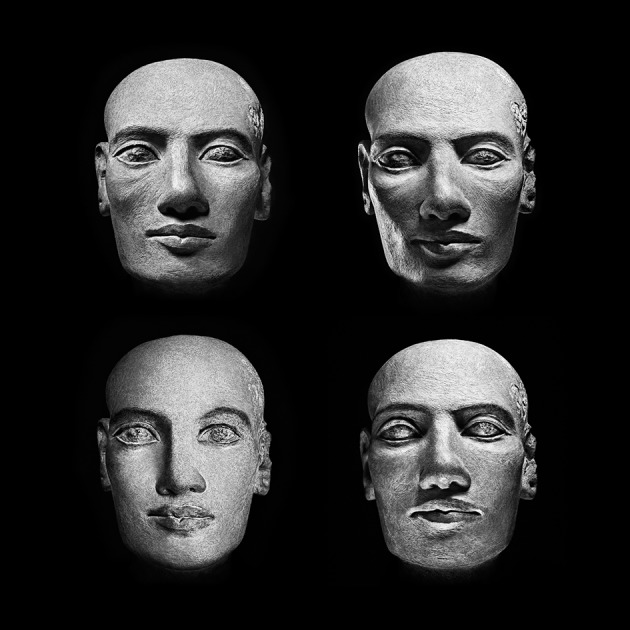
*****Head of Man*** Ca. 2289, known as ***Tête Salt***, painted limestone, estimated Middle Empire 18th–20th century BC possible provenance Karnack, High Egypt Department of Egyptian Antiquities Musée du Louvre, Paris, France, ***Humanum***® ***Paradeigma*** Square ***Q4*** C-1 dimension 1 to 1, 52.2 × 52.2 cm, Giorgia Fiorio © 2015**.

By means of a photo-mechanic process that fathoms the metamorphosis of the statuary form within the evolution of light, the *Humanum* process reveals to the viewer's eyes the endless transfiguration of the invisible aspects that surface over the sculpted matter. A selection of different images (modules) of the *head* of one same ideotype is thus organized by visual ensembles named *Paradeigma*. Each *Paradeigma* is composed by multiple modules—*one-to-one* size—of the original sculptural piece (Figures 2–4). To each module corresponds a negative silver *matrix*. Each *Paradeigma* constitutes an analog-digital structure encompassing multiple original negative matrixes; the quantity of modules is equal to a number multiplied by itself.

In the simultaneous comparison among different appearances of one same *physiognomy* (Gombrich, 1961, 1999; Hochberg, 1972) the alteration of the perceptual constancy provoked by the immutable sculptural evidence and the transformation of its human countenance induce a *vigilance* bond that, received by the retina, induces the re-positioning of the *focal fixation* (Arnheim, 1969b) and the iterative confrontation between different images. In this process, the transformation of the visible appearance is underpinned once again by the awareness of an intrinsic *recognition*. Such inner-outer dialectic discloses the *model*, unique to each viewer's eyes only, that is, the gesture of the thought that generates the subject's internal projection of perception and the *sign* of each vision.

At the height of the process that unhinged the semantic relationship between the observer and reality, the *Humanum*® project aims to re-transcribe the status of the human archetype—today merely seen as codified sign of a static *vestige* of the past—into the dialectic idea of origins as living heritage of the future. The project investigates the principle in which the original comes to light; it questions the invisible model of a representative *arché* disclosing an endless ‘apparent’ morphogenesis in the evolution of a luminous impulse.

The objective of the project is the transcription of such intuition into different languages and spheres of research. A *model* from an aesthetic canon to a scientific parameter and reverse, reconstituting a form of representation and exposition aiming to visualize different combinations of knowledge, interconnected by codified systems of signs and similar semantic foundations.^1^ *Humanum*® aspires to re-establish the *interiorization* of the visual experience according to the resonance of its dynamic principle and to elicit the awareness of the urgency of a scientific resilience in the evolution of technology.

## Author contributions

GF explores the condition between reality and appearance in the relationship between matter and figure. Questioning the consciousness of subjective experience beyond the manifestation of the visible, the project *Humanum*® reconsiders the dialectic between the terms vision-presence in the perception of human figuration according to the immaterial heritage of archaic human statuary. In progress in collaboration with Ca' Foscari University of Venice the *Humanum* the project encompasses so far a first archaic series in Greece at the National Archaeological Museum of Athens; one head piece (2nd CE) at the Acropolis Museum of Athens; an Egyptian stone head (18th–20th century BC) at the Musée du Louvre where it will have the first exhibition from June 2017 and a Sumerian marble head: the “Lady of Warka” (32nd century BC) at the National Museum of Iraq in Baghdad.

### Conflict of interest statement

The author declares that the research was conducted in the absence of any commercial or financial relationships that could be construed as a potential conflict of interest.

## Footnotes

^1^Since 2013 *Humanum*® project develops a parallel scientific research on related themes in collaboration with: Ca' Foscari University of Venice; L. Milano, Chair of History of the Antiquity and Near East Department of Humanities, Ca' Foscari University of Venice; G. Barbieri, Chair of History of Art and Cultural Heritage, Department of Philosophy and History of Art, Ca' Foscari University of Venice; M. Bergamasco, Chair of Robotics of Perception and M. Carrozzino, PhD in Virtual Reality and Computer Science of the *Percro* Laboratory, Scuola Superiore Sant'Anna; A. Schnapp, Chair of Classic Archaeology, National Institute of History of Art INHA, Paris; V. Stoichita, Chair of History of Art and Archaeology, Freiburg University (Switzerland); F. Vercellone Chair of Aesthetic Philosophy and Intra-University Morphology group of Turin University.
